# CO_2_ absorption into primary and secondary amine aqueous solutions with and without copper ions in a bubble column

**DOI:** 10.55730/1300-0527.3410

**Published:** 2022-02-23

**Authors:** Hamed YOUSEFZADEH, Cansu GÜLER, Can ERKEY, Erdal UZUNLAR

**Affiliations:** 1 Department of Chemical and Biological Engineering, Koç University, İstanbul, Turkey; 2 Department of Chemical Engineering, İzmir Institute of Technology, İzmir, Turkey

**Keywords:** CO_2_ absorption, carbon capture and storage, amine, copper, monoethanolamine, piperazine, homopiperazine, 2-piperidineethanol

## Abstract

Chemical absorption of CO_2_ into aqueous amine solutions using a nonstirred bubble column was experimentally investigated. The performance of CO_2_ absorption of four different primary and secondary amines including monoethanolamine (MEA), piperazine (PZ), 2-piperidineethanol (2PE), and homopiperazine (HPZ) were compared. The effects of initial concentration of amine, the inlet mole fraction of CO_2_, and solution temperature on the rate of CO_2_ absorption and CO_2_ loading (mol CO_2_/mol amine) were studied in the range of 0.02–1 M, 0.10–0.15, and 25–40 °C, respectively. The effect of the presence of copper ions in the amine solution on CO_2_ loading was also studied. By comparison of the breakthrough curves of the amines at different operational conditions, it was revealed that the shortest and longest time for the appearance of the breakthrough point was observed for MEA and HPZ solutions, respectively. CO_2_ loading of MEA, 2PE, PZ, and HPZ aqueous solutions at 25 °C, 0.2 M of initial concentration of amine, and 0.15 of inlet mole fraction of CO_2_ were 1.06, 1.14, 1.13, and 1.18 mol CO_2_/mol amine, respectively. By decreasing the inlet mole fraction of CO_2_ from 0.15 to 0.10, CO_2_ loading slightly decreased. As the initial concentration of amine and temperature decreased, CO_2_ loading increased. Also, the presence of copper ions in the absorbent solution resulted in a decrease in the CO_2_ loading of MEA and HPZ aqueous solutions. In case of PZ and 2PE amines, adding copper ions led to precipitation even at low copper ion concentrations.

## 1. Introduction

Global warming is the biggest environmental threat nowadays resulting in climate change with consequences such as rising sea levels, droughts, hurricanes, and extreme weather events [1]. Elevated concentration of greenhouse gasses (CO_2_, CH_4_, NOx) and especially of CO_2_ in the atmosphere in recent decades, is the main reason for global warming [2]. Power plants are responsible for more than 40% of CO_2_ emissions among which the coal-fired power plants release 73% of total CO_2_ emissions of fossil fuel-based power plants [3, 4]. The utilization of fossil fuels, especially coal, will continue because fossil fuels are still the cheapest options to produce electricity. Thus, it will be desirable to remove CO_2_ from the flue gas generated by power plants to stabilize the CO_2_ levels in the atmosphere [5].

Adsorption [6], absorption [7], membrane separations [8], cryogenic distillation [9], and chemical looping [10] are the technologies being investigated for CO_2_ capture from flue gas streams. Among these, post-combustion CO_2_ absorption by amine scrubbing seems to be a viable method already implemented in some industrial units, due to the desired efficiency of CO_2_ removal and ease of scale-up [11]. Also, amines are thermally regenerable solvents and have a strong affinity for CO_2_. Thus, the technology consists of absorption of CO_2_ by an aqueous amine solution at low temperature, generally, 40 °C, followed by regeneration of the amine solution at a higher temperature to release CO_2_ and recycle the fresh amine solution back to the absorber column [12,13]. The type of CO_2_ absorption using amines is chemical absorption during which dissolved CO_2_ molecules react with the nucleophilic nitrogen atom of amines forming a chemical bond between the nitrogen of amine and carbon of CO_2_. The physical and chemical properties of the CO_2_-H_2_O-amine system including viscosity, density, solubility of CO_2_, reaction rate, mass transfer rate, and regeneration heat determine the solvent performance of CO_2_ capture in the absorption/desorption system [11]. Amines are generally categorized into three main groups as primary, secondary, and tertiary amines [14]. Monoethanolamine (MEA) as a primary alkanolamine and piperazine (PZ) as a secondary amine have been frequently studied for CO_2_-amine-water systems in literature [15–21]. Especially, MEA aqueous solution is used as the absorbent in the industrial-scale CO_2_ removing systems [5, 22].

Huge energy consumption for solvent regeneration is the main drawback of amine-based CO_2_ capture systems. Electrochemical CO_2_ capture technology, which is a relatively new method under development, stands out by directly eliminating the high energy consumption in the MEA process since it is a process carried out at a low temperature [23]. In addition, electrochemical CO_2_ capture technology reduces electrical losses and prevents side reactions by precise control of potential. There are two main approaches to CO_2_ capture by electrochemical methods in the literature: (i) using electrochemical processes for both CO_2_ capture and CO_2_ release (overall approach), (ii) conventional methods for CO_2_ capture (e.g., MEA process) and CO_2_ release using the electrochemical method (hybrid approach) [24]. In the hybrid approach, formed CO_2_-amine complex is pumped to the anode of an electrochemical cell where metal ions (e.g., Cu(II)) are dissolved by the potential applied to the anode. These metal ions form a complex with the amine, allowing the amine-CO_2_ complex to release CO_2_. The resulting Cu(II)-amine complexes are pumped to the cathode in the electrochemical cell and the amine is liberated and recovered by reduction of the metal ion (reduced Cu(II) accumulates on the cathode as Cu(s)). Regenerated amine is then sent back to the absorption column. During the electrochemical regeneration, some copper-amine complexes stay in the aqueous phase [25] resulting in a decrease in CO_2_ loading compared to the copper ions free amine solution due to occupying some portion of free amines by the formation of the metal-amine complexes [26]. Nevertheless, the presence of copper ions and the interaction between copper ions and amines enable the modulation of CO_2_ loading in electrochemical CO_2_ capture systems.

In this study, the rate of CO_2_ absorption and CO_2_ loading of four primary and secondary amines including monoethanolamine (MEA), piperazine (PZ), 2-piperdineethanol (2PE), and homopiperazine (HPZ) in a lab-scale bubble column were investigated. Effect of process parameters including temperature, CO_2_ initial mole fraction, and amine concentration were studied by varying between 25–40 °C, 0.10–0.15, and 0.02–1 M, respectively. Also, the effect of the presence of copper ions in the amine solution on the CO_2_ loading was also studied by varying the concentration of copper ions between 0.02–0.2 M and compared to absorption without any copper ions. This was carried out for amines such as PZ, HPZ, and 2PE for the first time in the literature.

## 2. Materials and methods

### 2.1. Materials

Monoethanolamine (hereafter referred to as MEA) was obtained from the TÜPRAŞ R&D center. Piperazine (PZ), homopiperazine (HPZ), 2-piperidineethanol (2PE), and copper (II) nitrate were purchased from Sigma-Aldrich. CO_2_ and N_2_ gases (99.999%) were obtained from Air Liquide. Ultra-pure water (MilliQ, 18.2 MΩ) was used to prepare amine solutions at the desired amine concentration as the absorbent.

### 2.2. Absorption setup

**Figure 1:**
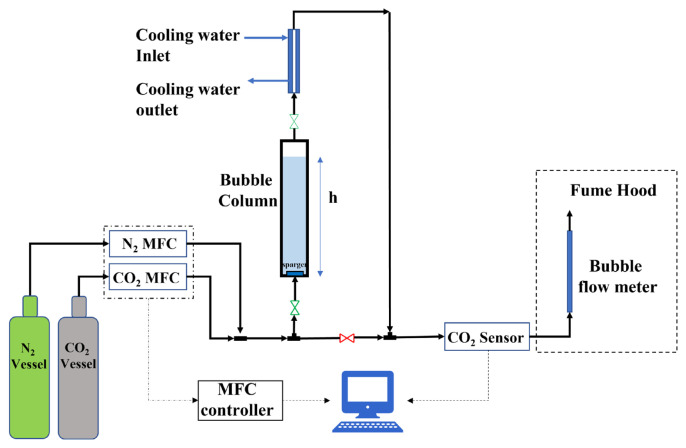
Experimental setup of CO_2_ absorption into amine solutions using a bubble column.

The schematic of the experimental setup of the CO_2_ absorption system used in this study is shown in Figure 1. A graduated cylinder with an internal diameter of 3.2 cm and a total volume of 280 mL was used as the bubble column. The top of this cylinder was plugged using a stopper with three holes to insert the tubing for inlet flue gas, the outlet gas, and a thermometer. Teflon tubes were used for all the gas lines. Prior to sending the simulated flue gas into the bubble column, an aqueous solution containing a certain amount of amine and MilliQ water was prepared and placed in the bubble column. The temperature of the column and amine solution was tuned to the desired temperature using a water jacket connected to a heating water circulator (Cole Parmer, Model 12108-15). Also, the gas mixture line was preheated by a water jacket up to the amine solution temperature. The temperature of the solution during the absorption experiments was recorded using a thermometer. The flue gas mixture was simulated by combining CO_2_ and N_2_ gas delivered by calibrated mass flow controllers (MFC, Teledyne Hastings HFC202). The mole fraction of CO_2_ in the gas mixture was adjusted by adjusting the volumetric flowrates of CO_2_ and N_2_ at 25 °C and atmospheric pressure. The flow rate of the gas mixture was kept constant at 250 mL/min. In the first stage, the entire inlet line was cleaned using a bypass line with pure nitrogen. Then, N_2_ gas was passed through the bubble column and the outlet line between the column and the CO_2_ sensor (GasLab, SprintIR®-W 100% CO_2_ sensor) was cleaned in the same way until the sensor showed zero mole fraction of CO_2_. Then, CO_2_ was added to the gas stream at a certain CO_2_ mole fraction and this time was recorded as the start time of CO_2_ absorption. The inlet gas line was sent through the stopper hole to the bottom of the bubble column and bubbled through an air stone before mixing it into the amine solution. CO_2_ in a gas mixture with a high concentration is captured by the amine solution in the column, and a gas mixture with zero or low CO_2_ concentration is obtained in the outlet. The amine and water vapor were removed from the outlet gas mixture using a condenser at 5 °C to prevent damaging the CO_2_ sensor by the amine and water vapor. Since the amount of amine vapor in the gas mixture leaving the condenser was negligible due to a very high Henry’s constant and vapor pressure of water was low at 5 °C, it was assumed that the outlet gas mixture consisted of only N_2_ and CO_2_. The mole fraction of CO_2_ in the outlet gas mixture was measured using the online CO_2_ sensor until the mole fraction reached the inlet mole fraction of CO_2_ (equilibrium condition). In every absorption experiment, the output CO_2_ concentration was plotted against time. This curve is called the breakthrough curve. Typically, in a breakthrough curve, a zero-outlet concentration of the compound of interest is observed up to a certain time after which the outlet concentration starts to increase. The time at which the outlet concentration reaches 5% of inlet concentration is called the breakthrough time. Also, saturation time is the time at which the inlet and outlet concentrations become identical. After this time, the system is in a liquid-gas thermodynamic equilibrium state.

### 2.3. Measurement of the rate of CO_2_ absorption

Mass balance for CO_2_ in the gas phase was performed to determine the rate of CO_2_ absorption from the gas phase into the liquid phase, as shown by equation (1).


(1) $$F_{{CO}_{2}(g),in}-F_{{CO}_{2}(g),out}-r_{{CO}_{2},(absorption)}=\frac{dN_{{CO}_{2}(g)}}{dt}$$

The accumulation term of equation (1) was rewritten in terms of the gas phase volume, concentration, and mole fraction of CO_2_ in the outlet gas mixture assuming a well-mixed solution in the column as follows:


(2) $$\frac{dN_{{CO}_{2}(g)}}{dt}=C_{out}.V_{g}.\frac{dy_{{CO}_{2}(g),out}}{dt}$$

Assuming an ideal gas mixture, the total concentration of the outlet gas mixture was calculated as follows:


(3) $$C_{out}=\frac{P_{out}}{RT_{out}}$$

where *P_out_*was equal to 1 atm and *T_out_*was taken as absorption column temperature, respectively.

The molar flow rate of CO_2_ in outlet, *P_CO_*__2_ (_*_g_*_), _*_out_* was rewritten as follows:


(4) $$F_{{CO}_{2}(g),out}=F_{N_{2}(g),out}\;\left(\frac{y_{{CO}_{2}(g),out}}{1-y_{{CO}_{2}(g),out}}\right)$$

The molar flow rate of nitrogen was assumed to be constant. Using equations (2), (3), and (4), the rate of absorption was derived from equation (1) as follows:


(5) $$r_{absorption}=F_{{CO}_{2}(g),in}-F_{N_{2},in}\;\left(\frac{y_{{CO}_{2}(g),out}}{1-y_{{CO}_{2}(g),out}}\right)-C_{out}.V_{g}.\frac{dy_{{CO}_{2}(g),out}}{dt}$$

The concentration of CO_2_ in the outlet gas mixture was measured and recorded by the sensor every 5 s. For each time the absorption rate was calculated using equation (5). The absorption rate data was plotted against time for each experiment.

### 2.4. Measurement of CO_2_ loading

The integral of the absorption rate versus time was equal to CO_2_ loading of the corresponding amine solution as shown in equation (6). The CO_2_ loading was calculated for all the experiments with different amines and process conditions. Effects of amine type (MEA, PZ, HPZ, 2PE), initial concentration of amine (0.02 M–1 M), inlet CO_2_ gas mol fraction (0.1 and 0.15), and amine solution temperature (25–40 °C) on CO_2_ loading were investigated.


(6) $$\alpha_{{CO}_{2}}=\int_{0}^{t_{final}}\left[F_{{CO}_{2}(g),in}-F_{N_{2},in}\;\left(\frac{Y_{{CO}_{2}(g),out}}{1-y_{{CO}_{2}(g),out}}\right)-C_{out}.V_{g}.\frac{dy_{{CO}_{2}(g),out}}{dt}\right]dt$$

## 3. Results and discussion

### 3.1. Breakthrough curves and CO_2_ loading

**Figure 2:**
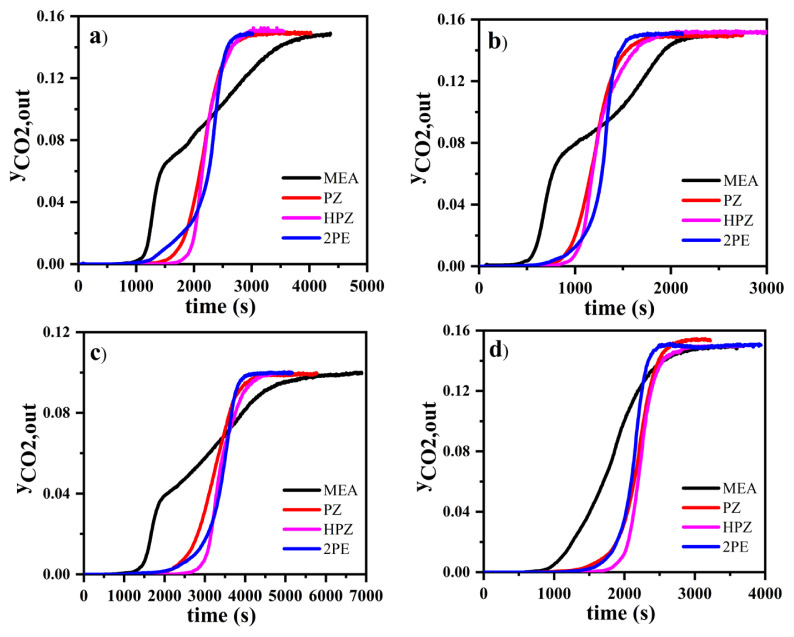
Breakthrough curves of CO_2_ absorption by amine solutions: initial concentration of amine, inlet mole fraction of CO_2_, and temperature were a) 0.2 M, 0.15, 25 °C; b) 0.1 M, 0.15, 25 °C; c) 0.2 M, 0.10, 25 °C; d) 0.2 M, 0.15, 40 °C, respectively.

According to the breakthrough curves given in Figure 2, the mole fraction of CO_2_ was zero at the outlet for a while, indicating that all the CO_2_ of the inlet stream was absorbed by the solution. After the breakthrough point was reached in each curve, the mole fraction of CO_2_ at the outlet stream started to increase more rapidly and finally reached the saturation point where the inlet and outlet mole fractions of CO_2_ were equal. The breakthrough curves for CO_2_ absorption of different amines at 25 °C shown in Figure 2a were obtained when the initial mole fraction of CO_2_ and initial concentration of amine were 0.15 and 0.2 M, respectively. The breakthrough time was approximately 1000, 1230, 1520, and 1700 s for MEA, 2PE, PZ, and HPZ, respectively. The breakthrough was observed after 2850, 3000, 3250, and 4300 s for 2PE, HPZ, PZ, and MEA, respectively. MEA was the amine with the fastest breakthrough but the latest to the saturation point. The effect of the initial concentration of amines on the rate of CO_2_ absorption and loading was investigated by lowering the initial concentration of amines from 0.2 M to 0.1 M while keeping other parameters unchanged. The breakthrough time and equilibrium state were obtained earlier at a low initial concentration of amines, as shown in Figure 2b. The breakthrough occurred at 500, 620, 790, and 825 s for MEA, 2PE, PZ, and HPZ, respectively, and equilibrium was observed after 1650, 1830, 2050, and 2340 s for 2PE, PZ, HPZ, and MEA, respectively. When the amine concentration was reduced to half, the breakthrough time was approximately two times faster. The breakthrough curves in Figure 2c were obtained by reducing the initial mole fraction of CO_2_ to 0.10 at 0.2 M amine concentration. When the gas mixture with a lower mole fraction of CO_2_ was delivered to the column, the breakthrough time increased (1150, 1695, 1915, and 2670 s for MEA, 2PE, PZ, and HPZ, respectively) compared to the case of 0.15 of CO_2_ mole fraction. The effect of temperature was investigated by increasing the temperature of the heating jacket from 25 to 40 °C while keeping the other parameters constant. The breakthrough times were obtained at 795, 1260, 1350, and 1685 s for MEA, PZ, 2PE, and HPZ, respectively. The breakthrough at 25 °C for MEA showed different behavior in comparison with the other amines. As shown in Figure 2a, the mole fraction of CO_2_ in the breakthrough curve of MEA solution at 25 °C increased rapidly starting from the breakthrough time up to a certain time followed by a slow increase in the mole fraction until the equilibrium condition was reached. For the experiments done with MEA at 40 °C, the equilibrium condition was obtained at a longer time range and the breakthrough time appeared earlier compared to other amines. Generally, the breakthrough time was obtained in shorter time ranges for MEA than other amines. In all experiments performed at 25 °C, the breakthrough time increased in the order of MEA, 2PE, PZ, and HPZ (MEA the fastest, HPZ the slowest). In the experiments carried out at 40 °C, the breakthrough time of PZ was ahead of 2PE, and the breakthrough times increased in the order of MEA, PZ, 2PE, and HPZ.

**Figure 3:**
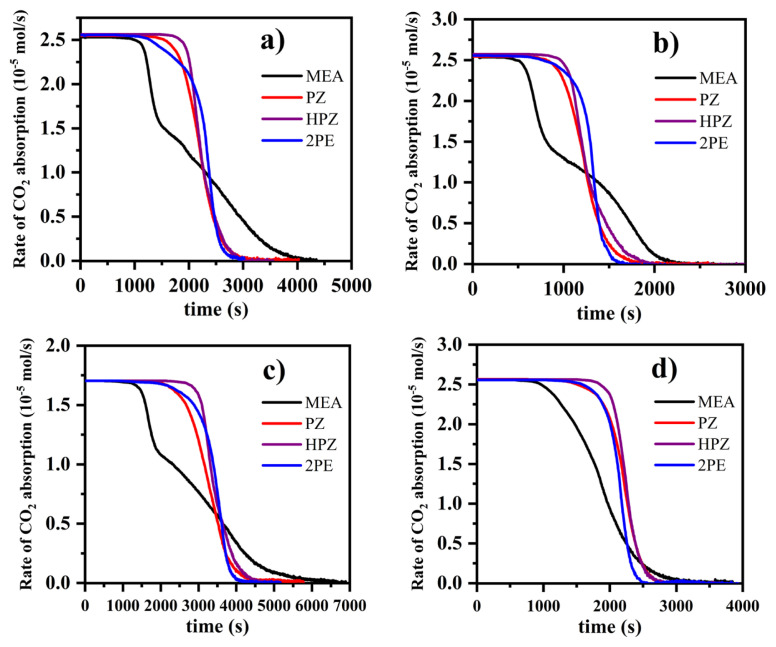
The rate of CO_2_ absorption into amine solutions: initial concentration of amine, inlet mole fraction of CO_2_, and temperature were a) 0.2 M, 0.15, 25 °C; b) 0.1 M, 0.15, 25 °C; c) 0.2 M, 0.10, 25 °C; d) 0.2 M, 0.15, 40 °C, respectively.

The rate of CO_2_ absorption for all experiments was calculated using equation (5) and plotted against the time, as shown in Figure 3. In all the curves shown, the rate of CO_2_ absorption was constant for a certain period of time since the mole fraction of CO_2_ at the outlet was zero during that period. After that period, the rate of CO_2_ absorption was observed to decrease. Zwitterion mechanism which was first proposed by Caplow [27] and reintroduced by Danckwert [28] was widely used to interpret the kinetic data of CO_2_ absorption into aqueous amine solutions. This mechanism consists of two main reactive steps. First, the nucleophilic nitrogen atom in the amine provides its electron pair to form a chemical bond with the electrophilic carbon atom of CO_2_. As a result, an unstable compound called zwitterion is produced. Then, the formed zwitterion reacts with any base compound in the solution such as OH^−^ and amine molecules (also H_2_O) to form carbamate. Using this mechanism, the rate of the CO_2_ absorption reactions is derived as a function of concentrations of amine, OH–, H_2_O, and CO_2_. According to the zwitterion mechanism, the rate of CO_2_ absorption is proportional to the amine concentration [27–29]. As the amine becomes depleted due to the reaction with CO_2_, the rate of CO_2_ absorption decreases and finally reaches the thermodynamic equilibrium state at which the rate of CO_2_ absorption becomes zero.

**Figure 4:**
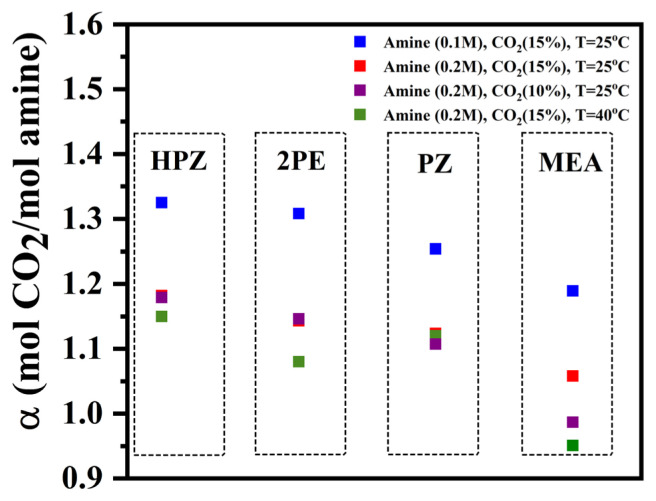
CO_2_ loading of HPZ, 2PE, PZ, and MEA amines (inlet mixed gas flow rate has been kept constant at 250 mL/min for all experiments).

The cumulative amount of absorbed CO_2_ was obtained by integration of absorption rate data versus time. Subsequently, the cumulative amount of absorbed CO_2_ was divided by the initial number of moles of amine in the system to obtain the CO_2_ loading of different amines. CO_2_ loading of HPZ, 2PE, PZ, and MEA at different process conditions is presented in Figure 4. According to this figure, in the absorption runs with inlet CO_2_ mole fraction of 0.15, HPZ showed the highest CO_2_ loading with 1.32 and 1.18 mol CO_2_/mol amine at 25 °C in both cases of 0.1 and 0.2 M of initial concentration of amines, respectively. CO_2_ loading in any CO_2_-H_2_O-amine system depends on the concentration of amine, temperature, and partial pressure of CO_2_. Even at a constant temperature at which the equilibrium constants of reactions are fixed, the equilibrium concentrations may change by changing the initial amounts of amine and CO_2_ partial pressure in the system [18, 30]. Here, as the initial concentration of amine decreased, the CO_2_ loading increased since a change in the initial concentration of the amine leads to the changes in the equilibrium concentrations of compounds in the vapor-liquid equilibrium system of CO_2_-H_2_O-amine [18,19]. The effect of inlet mole fraction of CO_2_ and absorption temperature were also investigated. When the inlet mole fraction of CO_2_ was reduced from 0.15 to 0.10, the CO_2_ loading remained approximately constant for HPZ, PZ, and 2PE, but decreased for MEA. Increasing the temperature from 25 to 40 °C at constant amine concentration (0.2 M) and inlet mole fraction of CO_2_ (0.15) resulted in a decrease in CO_2_ loading of MEA, 2PE, PZ, and HPZ aqueous solutions from 1.06 to 0.95, 1.14 to 1.08, 1.13 to 1.12, and 1.18 to 1.15, respectively. In conclusion, as the inlet mole fraction of CO_2_ increased and the initial concentration of amine and temperature decreased, the CO_2_ loading increased. The obtained results were compared to the literature. Jou et al. [31] reported that the solubility of CO_2_ in an MEA/H_2_O solution decreases by increasing the temperature and decreasing the CO_2_ partial pressure. They reported a solubility of about 0.6 mol CO_2_/mol amine at 25 °C, CO_2_ partial pressure of 11.8 kPa, and 30 mass percent of MEA (5 mol/L). However, the concentrations used in our study (0.1 and 0.2 mol/L) were much lower than 5 mol/L resulting in a higher CO_2_ loading which was in line with a trend that can be seen from the screening work conducted by Aronu et al. [30]. A similar result can be seen from another study, performed by Kim et al., which compared the CO_2_ absorption characteristics of aqueous solutions of diamines. They reported a CO_2_ loading of 0.775 and 0.839 mol CO_2_/mol amine for 30 mass percent of PZ and HPZ solutions (5 mol/L) at 40 °C and CO_2_ mole fraction of 0.3 in the inlet simulated gas stream. Here, they showed that increasing the temperature led to a decrease in the CO_2_ loading. The CO_2_ loading for lower amine concentrations was not reported [32]. Derks et al. reported that the CO_2_ loading into the aqueous solution of piperazine decreased with an increase in temperature and amine concentration. For example, they observed that the CO_2_ loading decreased from 1.02 to 0.98 mol CO_2_/mol amine when the temperature increased from 25 to 40 °C for an aqueous solution with amin concentration of 0.2 mol/L and CO_2_ partial pressure of approximately 10 kPa [33].

**Figure 5:**
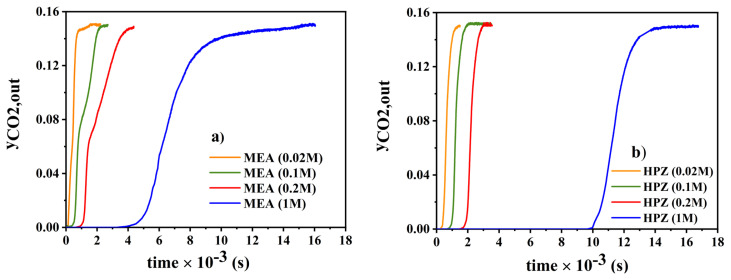
Effect of initial concentration of MEA and HPZ amines: a) MEA, b) HPZ (inlet mole fraction of CO_2_: 0.15, temperature: 25 ° C, the volumetric flow rate of the inlet gas mixture: 250 mL/min).

MEA and HPZ, the amines with the lowest and highest CO_2_ loading as shown in Figure 4, were chosen to be studied in a wide range of initial concentrations of amine ranging from 0.02 up to 1 M. To that end, the temperature of amine solutions, the total volumetric flow rate of the inlet gas mixture and the inlet mole fraction of CO_2_ were kept constant in all experiments at 25 °C, 250 mL/min and 0.15, respectively. According to the breakthrough curves shown in Figure 5, breakthrough times with respect to the initial amine concentrations of 0.02, 0.1, 0.2, and 1 M, were found to be 105, 500, 1000, and 4500 s for MEA and 240, 825, 1700, and 9980 s for HPZ, respectively. At all concentrations analyzed, breakthrough with MEA was faster than HPZ. Also, as the amine concentration increased, the differences between the breakthrough times for MEA and HPZ increased.

**Figure 6:**
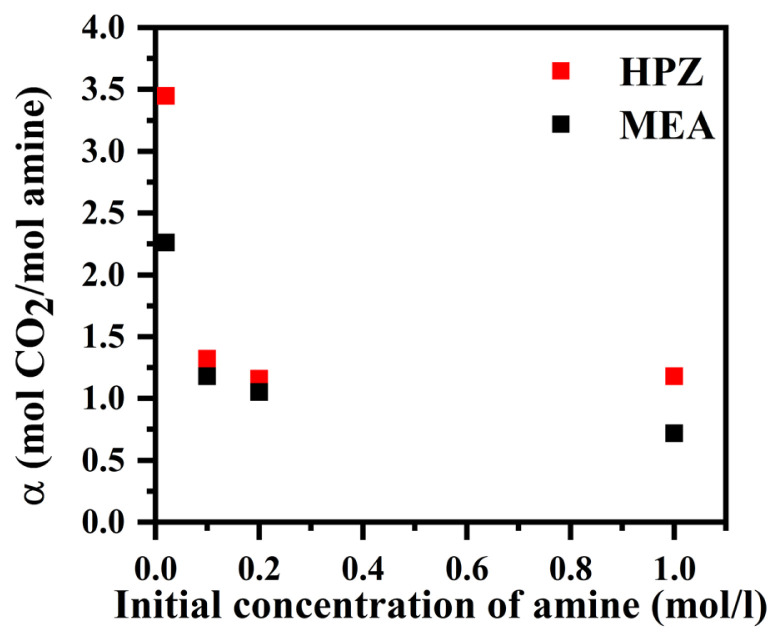
CO_2_ loading versus initial concentration of amines in HPZ and MEA solutions (inlet mole fraction of CO_2_: 0.15, temperature: 25 °C, volumetric flow rate of the inlet gas mixture: 250 mL/min).

CO_2_ loading was calculated according to Equation 5 and the results are presented in Figure 6. As the initial concentration of MEA increased from 0.02 to 1 M, the CO_2_ loading decreased from 2.25 to 0.72 mol CO_2_/mol amine. The results were similar to the literature. Aronu et al. reported that at an MEA concentration of 1 mol/L, the CO_2_ loading was obtained as 0.66 mol CO_2_/mol amine which was very close to the value obtained in our study (0.72 mol CO_2_/mol amine). Also, they showed that by further increasing MEA concentration from 1 to 5 mol/L, the CO_2_ loading decreased from 0.66 to 0.53 mol CO_2_/mole MEA. The curve of CO_2_ loading versus the amine concentration showed that using the amine concentrations lower than 1 mol/L led to a further increase of the CO_2_ loading, in line with results reported in our study. In addition, they showed that decreasing the CO_2_ partial pressure from 15 to 9.5 kPa led to a decrease in the CO_2_ loading for different MEA concentrations [18]. In experiments with HPZ, as the amine concentration increased from 0.02 M to 0.2 M, the CO_2_ loading decreased from 3.45 to 1.15 mol CO_2_/mol amine and remained almost constant by a further increase in the initial concentration of HPZ from 0.2 to 1 M.

### 3.2. pH of the solution

**Figure 7:**
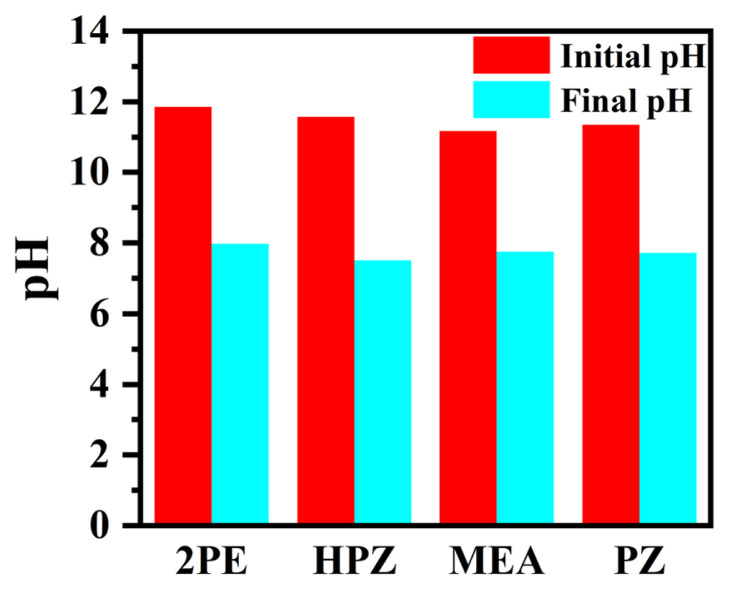
pH values of amine solutions at the beginning and at the end of CO_2_ absorption (initial amine concentration 0.2 M, input CO_2_ concentration 0.15, gas mixture flow rate 250 mL/min, temperature 25 °C).

According to the zwitterion mechanism [34, 35], the rate of CO_2_ absorption is affected by the concentration of the hydroxyl ion as well as the amine and CO_2_ concentration. In this mechanism, as the concentration of the hydroxyl ion decreases, the rate of CO_2_ absorption also decreases. For this purpose, the pH values of the amine solutions at the beginning and the CO_2_ saturation point were measured in the experiments performed at 25 °C, 0.2 M of initial amine concentration, and 0.15 of inlet CO_2_ mole fraction. The results are presented in Figure 7. Amine solutions were initially basic with pH values above 11. Table 1 shows the reactions which are expected to take place in the CO_2_-amine-water system during the absorption/desorption [31, 34, 36, 37]. pH values above 11 of amine solutions before feeding CO_2_ was due to protonation of amine shown by reaction R4 in Table 1, consuming hydronium ions. The pH values decreased after the solutions were fed with CO_2_. According to reactions in Table 1, as the solution was fed with CO_2_, the reaction R2 goes in the forward direction producing bicarbonate ions and hydronium ions. The produced bicarbonate ions are consumed by amine in the forward reaction R5. This decreases the bicarbonate ion concentration forcing reaction R2 to proceed in the forward direction producing more bicarbonate ions as well as hydronium ions. A portion of hydronium ions is also produced by forward reaction of R3. As a result, the increase in hydronium ion concentration, as well as a decrease in hydroxyl ion, causes the pH value of the solution to decrease. Initial and posttreatment pH values decreased from 11.86 to 7.98, 11.57 to 7.51, 11.17 to 7.76, and from 11.35 to 7.72 for 2PE, HPZ, MEA, and PZ, respectively. Since the solution came to thermodynamic equilibrium after the treatment, the solution was not completely neutralized since the unreacted amines stay in the solution even if their concentration was small.

### 3.3. Effect of copper ions

**Figure 8:**
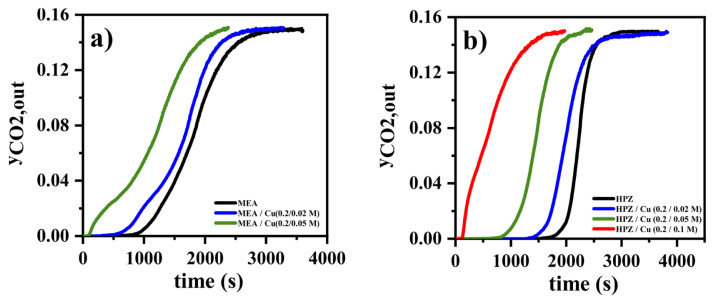
Copper ion effect (0.2 M of initial concentration of amine, 0.15 of inlet mole fraction of CO_2_, 40 °C of temperature) (No data for 0.2 M MEA solution at 0.1 M Cu(II) concentration was obtained since precipitation was observed).

In electrochemical amine regeneration systems, the amine-CO_2_ complex is pumped to the anode of an electrochemical cell where the metal ions (e.g., Cu(II)) are dissolved by the potential applied to the anode. These metal ions compete with CO_2_ to form a complex with the amine, allowing the release of CO_2_. The obtained metal(II)-amine complexes are pumped to the cathode in the electrochemical cell to reduce the metal ion and regenerate the amine (reduced metal ions accumulate in the solid metal form on the cathode). Copper is the most preferred metal to be used in these electrochemical cells [24]. The presence of copper ions in the regenerated amine solution affects the CO_2_ loading of the solution. For this reason, the CO_2_ loading of amine solutions with different copper ion concentrations was measured. Copper(II) nitrate was added to the solutions to prepare the certain concentrations of copper ions in the solutions. Precipitation was observed for 2PE and PZ amines as copper ions were introduced into the solution. For this reason, only aqueous solutions containing MEA and HPZ were subjected to CO_2_ absorption in the presence of copper ions. MEA and HPZ solutions with copper ions in a concentration range of 0.02–0.1 M were prepared. The initial concentration of amine, the inlet mole fraction of CO_2_, inlet gas mixture volumetric flow rate, and temperature of the solution were kept constant at 0.2 M, 0.15, 250 mL/min, and 40 °C, respectively. According to Figures 8a and b, as the copper concentration increased in MEA and HPZ solutions, the breakthrough time decreased.

**Figure 9:**
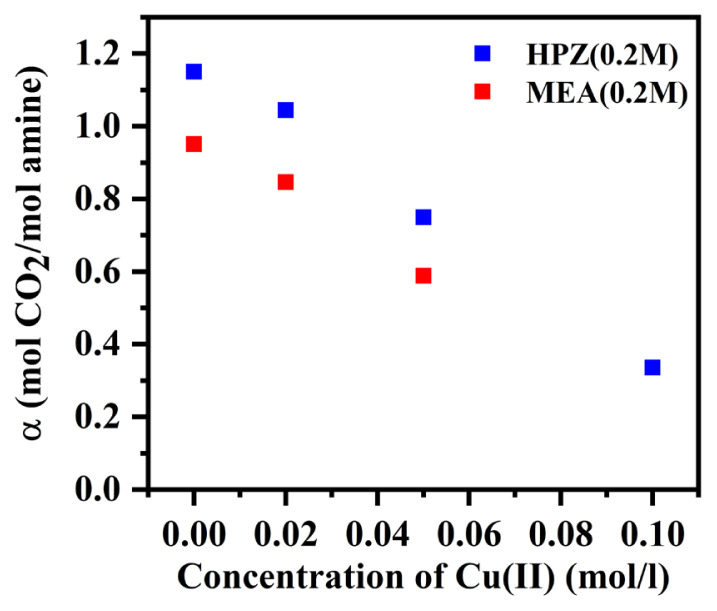
Change of CO_2_ loading with Cu(II) concentration for HPZ and MEA solutions. (No data for 0.2 M MEA solution at 0.1 M Cu(II) concentration was obtained since precipitation was observed).

Figure 9 shows the CO_2_ loading of each case calculated by integration of the absorption rate of CO_2_ versus time, as explained in section 2.3. It was found that as the concentration of copper (II) ions increased, CO_2_ loading for both HPZ and MEA decreased significantly from 1.15 (0 M copper) to 0.33 mol CO_2_/mol HPZ (0.1 M copper), and from 0.95 (0 M copper) to 0.59 mol CO_2_/mol MEA (0.05 M copper), respectively. Reactions R7, R8, R9, and R10 shown in Table 2 indicate that copper ions react with one, two, three, or four amines, respectively, and form different types of copper (II)-amine complexes [26]. Only a very small fraction of the copper(II)-amine complexes can form free amines as a result of the reverse reaction. Thus, the concentration of free amines in this system is smaller compared to that of copper-free amine solutions since most of the copper(II)-amine complexes are not regenerated as free amine due to higher equilibrium constants of copper (II)-amine complex formation reactions [26, 38]. As a result, CO_2_ loading in systems containing copper (II) ions decreases. According to Figure 9, the decreases in CO_2_ loading due to the presence of copper ions were observed for both MEA and HPZ. Especially the large change in CO_2_ loading in case of HPZ is promising for electrochemical amine regeneration.

## 4. Conclusion

The process of CO_2_ capture by amine-based chemical absorption routes has high CO_2_ removal efficiency and is easy to scale up. The physical and chemical properties of amine-H_2_O-CO_2_ systems affect the CO_2_ removal efficiency. Among them, viscosity, density, solubility of CO_2_, type of amine, rate of reaction, rate of mass transfer, and heat of regeneration are the important factors affecting the CO_2_ removal efficiency. In this study, the effect of type of amine on CO_2_ loading at different amine concentrations, inlet mole fractions of CO_2_, and temperatures was investigated. Aqueous solutions of four different amines including MEA, 2PE, PZ, and HPZ were used as the absorbent for chemical absorption of CO_2_ using a nonstirred bubble column. CO_2_ loading of MEA, 2PE, PZ, and HPZ aqueous solutions at 25 °C, 0.2 M of initial concentration of amine, and 0.15 of inlet mole fraction of CO_2_ was 1.06, 1.14, 1.13, and 1.18 mol CO_2_/mol amine, respectively. Thus, the highest CO_2_ loading was observed for HPZ among the studied amines. By decreasing the inlet mole fraction of CO_2_ from 0.15 to 0.10, the CO_2_ loading slightly decreased. As the initial concentration of amine decreased to 0.1 M, the CO_2_ loading increased to 1.19, 1.30, 1.25, 1.32 mol CO_2_/mol amine for MEA, 2PE, PZ, and HPZ, respectively. Also, increasing the temperature up to 40 °C decreased the CO_2_ loading to 0.95, 1.08, 1.12, 1.15 mol CO_2_/mol amine for MEA, 2PE, PZ, and HPZ, respectively. The CO_2_ removal efficiency of the interested amine solutions in presence of copper ions was also investigated. The results demonstrated that the addition of copper ions to the fresh amine solution leads to a decrease in the CO_2_ loading of MEA and HPZ aqueous solutions. Increasing the concentration of copper ions from 0 to 0.05 M resulted in a decrease in CO_2_ loading at 40 °C, from 1.15 to 0.75 and 0.95 to 0.59 mol CO_2_/mol amine for HPZ and MEA, respectively. Further increase in the concentration of copper ions to 0.1 M led to precipitation in MEA solution and a further decrease in CO_2_ loading to 0.34 mol CO_2_/mol amine for HPZ solution. However, in the case of PZ and 2PE amines, adding copper ions led to precipitation even at low copper ion concentrations.

## Figures and Tables

**Table 1:** Reactions occurring in the liquid phase during absorption of CO_2_ by an amine solution [30, 32–34].

Reaction	Reaction number
$2H_{2}O\overset{k1}{\underset{k-1}{⇄}}H_{3}O^{+}+{OH}^{-}$	(R1)
${CO}_{2}+2H_{2}O\overset{k_{2}}{\underset{k_{-2}}{⇄}}{HCO}_{3}^{-}+H_{3}O^{+}$	(R2)
${HCO}_{3}^{-}+H_{2}O\overset{k_{3}}{\underset{k_{-3}}{⇄}}CO_{3}^{2-}+H_{3}O^{+}$	(R3)
$R_{1}R_{2}NH+H_{3}O^{+}\overset{k_{4}}{\underset{k_{-4}}{⇄}}R_{1}R_{2}NH_{2}^{+}+H_{2}O$	(R4)
$R_{1}R_{2}NH+{HCO}_{3}^{-}\overset{k_{5}}{\underset{k_{-5}}{⇄}}R_{1}R_{2}{NCOO}^{-}+H_{2}O$	(R5)
$R_{1}R_{2}{NCOO}^{-}+H_{3}O^{+}\overset{k_{6}}{\underset{k_{-6}}{⇄}}R_{1}R_{2}{NH}^{+}{COO}^{-}+H_{2}O$	(R6)

**Table 2:** Reactions of Cu with amine in Cu–amine–CO_2_–H_2_O system [26].

Reaction	Reaction number
*Cu*^2+^ *R*_1_*R*_2_*NH*⇆*Cu*(*R*_1_*R*_2_*NH*)^2+^	(R7)
${Cu}^{2+}+2R_{1}R_{2}NH⇄Cu{\left(R_{1}R_{2}NH\right)}_{2}^{2+}$	(R8)
${Cu}^{2+}+3R_{1}R_{2}NH⇄Cu{\left(R_{1}R_{2}NH\right)}_{3}^{2+}$	(R9)
${Cu}^{2+}+4R_{1}R_{2}NH⇄Cu{\left(R_{1}R_{2}NH\right)}_{4}^{2+}$	(R10)

**Table 3:** Nomenclature

*N* *_i_*	number of moles of species *i*, mol
*F* *_i_*	molar flow rate of species *i*, mol/s
*r_i,_*_(_*_absorption_*_)_	rate of absorption of species *i*, mol/s
*T*	time, s
*C* *_i_*	concentration of species *i*, mol/m^3^
*V* *_g_*	volume of gas phase, m^3^
*y* *_i_*	mole fraction of species *i*, mol/mol
*P*	pressure, Pa
*T*	temperature, K
*R*	gas constant, J/(mol.K)
*α* *_CO_*_*_2_*_	CO_2_ loading, mol CO_2_/mol amine
